# Patient-related constraints on get- and be-passive uses in English: evidence from paraphrasing

**DOI:** 10.3389/fpsyg.2013.00848

**Published:** 2013-11-12

**Authors:** Dominic Thompson, S. P. Ling, Andriy Myachykov, Fernanda Ferreira, Christoph Scheepers

**Affiliations:** ^1^Department of Psychology, Institute of Neuroscience and Psychology, University of GlasgowGlasgow, UK; ^2^Department of Psychology, Northumbria UniversityNewcastle, UK; ^3^Department of Psychology, Institute for Mind and Brain, University of South CarolinaColumbia, SC, USA

**Keywords:** paraphrasing, get-passive, voice, information structure, transitivity, passivization

## Abstract

In English, transitive events can be described in various ways. The main possibilities are active-voice and passive-voice, which are assumed to have distinct semantic and pragmatic functions. Within the passive, there are two further options, namely be-passive or get-passive. While these two forms are generally understood to differ, there is little agreement on precisely how and why. The passive Patient is frequently cited as playing a role, though again agreement on the specifics is rare. Here we present three paraphrasing experiments investigating Patient-related constraints on the selection of active vs. passive voice, and be- vs. get-passive, respectively. Participants either had to re-tell short stories in their own words (Experiments 1 and 2) or had to answer specific questions about the Patient in those short stories (Experiment 3). We found that a given Agent in a story promotes the use of active-voice, while a given Patient promotes be-passives specifically. Meanwhile, get-passive use increases when the Patient is marked as important. We argue that the three forms of transitive description are functionally and semantically distinct, and can be arranged along two dimensions: *Patient Prominence* and *Patient Importance*. We claim that active-voice has a near-complementary relationship with the be-passive, driven by which protagonist is *given*. Since both *get* and *be* are passive, they share the features of a Patient-subject and an optional Agent by-phrase; however, *get* specifically responds to a Patient being marked as important. Each of these descriptions has its own set of features that differentiate it from the others.

## Introduction

A transitive event is one involving two participants: an Agent, the “doer” of the action, and a Patient, the person or thing that “undergoes” the action^1^. In English such events can be described in Active-voice or Passive-voice. While active is the canonical form, the passive serves distinct specific functions.

The function of the passive-voice in general is to allow focus to be directed toward a specific element, in a manner similar to other topicalizing constructions such as clefting (Keenan and Dryer, 2006). In the case of the passive, it is the Patient of a described event that is elevated above other elements; that is, the syntactic prominence of the Patient in the passive is utilized in communicating some form of significance. As Keenan and Dryer (2006) note, this has the complementary effect of allowing the backgrounding of another element, namely the Agent. While the Agent occupies subject position in active-voice descriptions, in the passive it is reduced to an agentive by-phrase, as in *Mary was hired by the manager*. The prominence of the Agent can be further reduced by removing the by-phrase entirely, as in *Mary was hired*.

It is notable that the passive is further divided, providing the options of be-passive and get-passive. Some have dismissed these two versions as equivalent, both syntactically (Chomsky, 1981) and semantically (Weiner and Labov, 1983). However, literature from both Linguistics and Psychology points to these two passive-types having their own distinct uses, structures, and connotations, as we will discuss in the subsequent sections.

Many distinguishing factors have been suggested, from the nature of the verb or the described event, to the language modality or variety of English. The passive Patient is frequently cited, though there are widely differing opinions as to the precise attribute of the Patient motivating the use of one passive-type over the other. Here, we aim to address this situation by experimentally investigating the role of the Patient, and how that role may differ between the two passive-types; *get* and *be*.

We will show that be-passives and get-passives have complementary responses to various Patient-related attributes: *get* displays a preference for important or focussed Patients, regardless of which protagonist is given, while *be* displays preference for a given Patient, regardless of any marked importance on Agent or Patient.

We will argue that active-voice, be-passive, and get-passive may be conceptualized as three distinct forms of transitive description, rather than primarily involving a voice distinction (active or passive), with passive-type (*be* or *get*) being a minor and largely syntactic matter.

It is well-established that the passive-voice is the sub-dominant form for descriptions of transitive events in English. It has also been found that passives are more likely in written as opposed to spoken language. Chafe (1982) notes that passives are as much as five times more common in the written modality. Likewise, in a study utilizing the BNC, Brown, Switchboard, and Wall Street Journal corpora, Roland et al. (2007) found that passives were less common in spoken data. Biber (1993) notes that passives are much more common in scientific writing than in spoken conversation or fictional writing.

Mair and Leech (2006) examined British and American English corpora: LOB, F-LOB, Brown, and Frown. They report a decline in the use of be-passives over time, as well as a rise in the use of get-passives. Interestingly, this pattern is observed in both British and American English. However, it does not seem to be the case that the be-passive is simply losing ground to the get-passive, since in raw numbers the fall of *be* far outweighs the rise of *get*. The authors relate this change to an apparent shift in written English toward the norms of spoken language.

While passives are less common than actives, *within* the passive it is *get* that is least frequent. Carter and McCarthy (1999) considered the CANCODE spoken English corpus and found just 139 instances of get-passive from a sample of 1.5 million words. Xiao et al. (2006) also note far fewer examples of get-passives than be-passives in both F-LOB and BNC.

Collins (1996) reports that get-passives are less common in formal language settings. This is compatible with Biber et al. (1999) who claim that get-passives are almost exclusively found in conversation. Likewise, Mindt (2000) reports that get-passives are mostly found in spoken language.

The most frequently reported feature of the get-passive in corpus literature is that it tends to appear more frequently without an agentive by-phrase. Collins (1996) as well as Carter and McCarthy (1999) found that more than 90% of get-passives did not include a by-phrase. Mindt (2000) reports 82%; while Rühlemann (2007) states that get-passives included a by-phrase with a frequency of just 0.079 per 1000 utterances. Xiao et al. (2006), who looked at F-LOB and BNC, state that agentless passives were the most common form for both *get* and *be*, with no strong difference between them in terms of by-inclusion. However, Guoliang and Lei (2010) report fewer by-phrases in get-passives than in be-passives for both British (BNC) and American (COCA) English.

With regard to the specific function and meaning of get-passives, there are numerous partially conflicting or even completely opposing claims. They range from *get* and *be* equivalence (e.g., Weiner and Labov, 1983) or non-equivalence (e.g., Lasnik and Fiengo, 1974; Chappell, 1980; etc.), to specifics such as get-passives communicating primarily negative outcomes (e.g., Sawasaki, 2000) or equally communicating both negative and positive outcomes (e.g., Sussex, 1982; Givón, 1993; Sasaki, 1999). Recent work (Thompson and Scheepers, 2013) has suggested a theoretical model to account for be-passive and get-passive syntax and semantics in a more parsimonious manner by way of a shared structural component.

Meanwhile, the majority of experimental data on get-passives is concerned with child language use, rather than adult use. As a result, the research questions addressed are primarily linked to development, such as the age at which children understand passives or can be primed to use passive-voice vs. active-voice (see Thompson, 2012 for a summary). With so much unresolved regarding how get-passive usage and semantics differ from the be-passive, there remains a great demand for further investigation.

Within the discordant arguments in the literature that try to separate the get-passive from the canonical be-passive, one aspect that is often discussed is the nature or role of the Patient. While there is not a great deal of agreement on the specifics, the Patient is frequently accorded some manner of “special” role or focus in the get-passive. It is the more prominent of these Patient attributes that we address in the present work.

Hatcher (1949) asserts that the Agent in the get-passive holds a subordinate role, which, as a result, gives the primary role to the Patient. For Palmer (1974), *get* communicates an action as well as the state that results from it, implying a strong transitivity or change of state for the Patient.

Several authors use the term “affected,” though not consistently. In general, if an entity is affected, it is understood as undergoing some experience resulting from the action described by the main verb. Authors including Sasaki (1999) believe that the get-passive suggests greater subject (i.e., Patient) affectedness. In a similar vein, Carter and McCarthy (1999) suggest that the get-passive has a tendency to focus on the event itself, along with the way in which the event impacts the Patient.

Cameron (1990) suggests that, to warrant the use of *get*, it is not sufficient for the Patient to simply be affected; rather the Patient must be “materially affected,” that is, the event must involve a material action, as opposed to an emotion or thought. Orfitelli (2011) even claims that “the ‘affectedness’ requirement is so strong that predicates that do not affect their internal argument are typically illicit with the get-passive, although they are allowed in the be-passive.” The latter two claims are actually not well-supported by our own corpus searches. A brief interrogation of COCA (COCA, 2012) or BNC (BNC, 2012) indicates that get-passives are commonly used with “non-affecting” verbs, including Orfitelli's specific example. Conducting a search in COCA for “[get] seen” (i.e., “lemma GET immediately preceding the exact form seen”), returns results such as “14 billion videos get seen on YouTube every month,” “it might make it slightly easier for me to get seen,” “it would not get seen by anyone,” etc. This precise and constrained search returns 34 tokens, with comparable results for numerous other “non-affecting” verbs, such as *watched* or *spotted*. Clearly, get-passives are not impossible with such verbs.

Get-passives are also widely conceptualized as communicating a sense of adversity for the Patient of the action. For some, adversity can only refer to events with negative outcomes, for example Sawasaki (2000), who suggests this tendency is most apparent with human protagonists. Carter and McCarthy (1999) also suggest that get-passives communicate adversity; however, they specify that the adversity is interpreted by the speaker, rather than by the actual Patient of the action.

Other authors hold a broader view of adversity, taking it to refer equally to both positive and negative outcomes (Chappell, 1980; Sussex, 1982; Siewierska, 1984; Givón, 1993; Gronemeyer, 1999; Sasaki, 1999). The adversity may be due to a sense of struggle; McIntyre (2005) describes the get-passive as suggesting “the result is hard to attain.” Sussex (1982) suggests that, while get-passives can communicate both positive and negative events, they have more semantic flexibility when they are negative.

Many authors note some implication of initiative, control, or responsibility in get-passive descriptions (among many others, Hatcher, 1949; Lakoff, 1971; Barber, 1975; Givón and Yang, 1994; Downing, 1996; Sasaki, 1999). A similar feature is noted by Vanrespaille (1991), who suggests that “resultativeness” (an action that leads to a result that cannot be undone) is a major feature of the get-passive, and also that the Patient is at least partly responsible for the occurrence of the action. Arrese (1999) notes this “partial responsibility” of the Patient. Sussex (1982) claims that get-passives can imply varying degrees of purposefulness, blame, and responsibility, as well as other meanings.

Lasnik and Fiengo (1974) conceptualize the semantics of control in a rather more complementary manner between the two passive-types. The authors suggest that a passive sentence formed with *get* implies Patient control, while the same sentence formed with *be* implies Agent control. For example, they state that (1) implies that the Patient, John, intended to cause the event; while in (2), it is the Agent, Mary, who intended to cause it.

1. John got fouled by Mary on purpose
2. John was fouled by Mary on purpose

Givón (1993) provides comparable examples.

According to Cameron (1990), the Patient's responsibility in the get-passive comes from association with causative *get* (as in *He got her fired*), not from any inherent feature of get-passive semantics. A similar claim is made by Hatcher (1949), suggesting that the responsibility is extended from the overt responsibility of reflexive *get* (as in *He got himself fired*).

Another potentially highly relevant constraint on passivation as a whole, is information structure. In the linguistic literature, information structure is a term that is used to cover numerous related concepts such as topicality, givenness, and focus within a wider discourse. These concepts are also used with varying definitions. Birner and Ward (1998) generalize givenness as the level of availability of information. For our purposes we consider givenness to represent what is old or new to a hearer: information that is old is *given*; it is the entity that is the topic of a sentence or discourse, and has been *available* for longer. Once an entity has been established as given (for example, *A new delivery boy arrived at our house*.), any time that it re-occurs in the same discourse, it is likely to be referred to with a pronoun (e.g., *He …*).

This distinction of given vs. new is important here because given referents in a discourse tend to assume a prominent syntactic role, typically the subject position in English. Therefore, the givenness or newness of the Agent or Patient of an action should influence the selection of active-voice vs. passive-voice, with a given Patient increasing the use of passives. Relatedly, in a study by Meints (2003), scene descriptions were prompted via Patient-directed questions. This established the Patient as *referentially given* (Gundel, 1988, 1999), and resulted in passive responses being the most frequent (60.8% overall).

In this paper, we aim to contribute experimental data to these discussions. We use a paraphrasing task, since givenness and focus manipulations can be achieved through basic linguistic attributes, such as clefting as discussed below. Using linguistic rather than visual stimuli (e.g., picture description) avoids any influence of visual prominence, and allows a natural way to introduce one protagonist before the other. We also utilize Patient-directed questions, but with additional implications regarding the Patient's role in the event.

First, we test the impact of *Givenness* on the description of transitive events. Experiment 1 considers the effects of given information, vs. new information, on the rate of passive production, establishing baseline probability levels for active- vs. passive-voice (and the various passive-types) as a function of information structure.

Following this, we combine *Givenness* with additional manipulations in order to investigate specific aspects of the Patient in be-passives and get-passives. Experiment 2 considers a general sense of importance or focus applied to the Patient established via clefting (which is another type of topicalizing construction; see above), while Experiment 3 investigates potential effects of implied blame, control, or responsibility on the part of the Patient, which we take together as forms of “Patient agency.”

## Experiment one

### Introduction

Experiment 1 manipulated only *givenness* of the Agent or Patient. This allowed us to establish baseline levels of active-voice vs. passive-voice, and be-passive vs. get-passive, as a function of information structure. This serves as a comparison for the findings of Experiments 2 and 3, in which additional experimental factors are manipulated.

### Participants

Twenty-four native English speakers (age 18–56, mean age 24; 29% males) were tested in individual sessions, with each lasting approximately 30 min. They all gave informed consent and received subject payment or course credits for their participation. All participants were recruited through the University of Glasgow's subject database^2^.

### Stimuli

Twenty-four short stories were created (as in 3), each consisting of two sentences. The second sentence described a transitive event in active-voice, while the first provided a preamble to the event. One of the two protagonists of the event, namely either the Patient (3a) or the Agent (3b) of the event in the second sentence, was introduced in the first sentence and was therefore *given*; i.e., when participants encountered the sentence describing the transitive event, one of the two protagonists was already known (given), while the other was not previously known (new). This constituted the conditional manipulation *information status*.

(3a) *The cowboy rode across the desert into the small dusty town*.
  *When he arrived a thief attacked him*.
(3b) *The thief made her way through the sandstorm to a small town*.
  *When she arrived she attacked a cowboy*.

The transitive description was always in active-voice, meaning that any increase in passive production would be motivated by the experimental factor. This produced a two-level design with two Information Status levels. A full list of materials is given in the appendix (Table A1).

### Procedure

All experiments within this paper employed a paraphrasing paradigm. In this instance, the 24 (items) × 2 (conditions) were assigned to 2 separate lists such that each item appeared precisely once per file, and in a different condition in each of the two files, using a Latin square. This resulted in twelve items per condition per file, ensuring an equal frequency of each condition in each file. Further to the 24 critical items per file, 50 filler items were also included. These fillers varied structurally with various features to distract participants from the intentions of the study. This gave a total of 74 trials per file.

The task was presented on a 12· LCD monitor running at 60 frames per second and was run using SR Research Experiment Builder. Participants interfaced with the task using a keyboard. The spacebar was used to advance through trials and to advance from one screen to the next within trials. A short practice session preceded the main experiment to familiarize participants with the procedure and type of sentences they would be encountering in the experimental trials and fillers.

**Figure 1:**
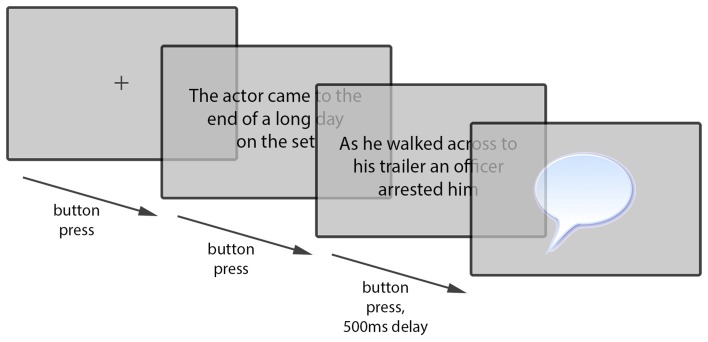
**Example of paraphrasing trial procedure**.

Trials proceeded as shown in Figure 1. They always began with a central fixation cross. The next screen displayed the preamble sentence in the center of the screen. After reading this aloud, participants pressed the spacebar to advance. The next screen displayed the transitive event sentence in the center of the screen. Again, this sentence was read aloud. After a brief (500 ms) pause, the next screen displayed a prompt for participants to retell the transitive event described in the second sentence in their own words. They responded to this out loud, and then pressed the spacebar to end the trial and begin the next.

Participants' spoken responses were audio recorded on the experimenter computer and coded for “voice” at three levels: active (transitive sentence in active-voice, with the subject referring to the Agent and the direct object to the Patient of the critical event), be-passive (sentence in passive-voice using a form of *be* as auxiliary verb and with the subject referring to the Patient of the critical event), or get-passive (sentence in passive-voice using a form of *get* and with the subject referring to the Patient of the critical event). Passive responses were also coded for agentive by-phrase inclusion (i.e., whether the sentence contained a prepositional phrase headed with *by* that referred to the Agent of the critical event). Each participant was presented with one of the two files (74 trials), which were split into 3 blocks, allowing for breaks to maintain attentiveness.

### Results

In this experiment we were concerned with the production of syntactic alternatives, thus it was deemed necessary for participants to demonstrate the availability of at least two forms. Those who did not produce any passive responses were discarded and further participants were tested to replace them; in this instance, only one participant was replaced. Following this, the data were filtered before analysis. Responses that were not transitive were coded as errors and were discarded. Less than 2% of the data were excluded.

Table 1 provides the raw descriptive counts for five response types (active-voice, be-passive with or without an agentive by-phrase, get-passive with or without a by-phrase) in each condition (given Agent/given Patient).

**Table 1:** **C*ross-tabulation* results giving raw counts of responses (AV, active-voice; B0, be-passive without by-phrase; BB, be-passive with by-phrase; G0, get-passive without by-phrase; GB, get-passive with by-phrase) in each of the two conditions (given Agent, given Patient)**.

**Condition**	**Response Type**				
	**AV**	**B0**	**BB**	**G0**	**GB**
Given Patient	129 (0.46)	21 (0.07)	130 (0.46)	0 (0.00)	3 (0.01)
Given Agent	221 (0.78)	12 (0.04)	48 (0.17)	0 (0.00)	1 (0.00)
Total	350 (0.62)	33 (0.06)	178 (0.32)	0 (0.00)	4 (0.01)

*Rounded probabilities per condition (respectively per Total) are shown in parentheses*.

Table 1 indicates a strong overall preference for active-voice responses, especially in the presence of a given Agent. Get-passive responses were extremely rare, always including an agentive by-phrase when they occurred. While be-passive responses were less frequent than active-voice responses overall, in the presence of a given Patient, be-passives became slightly more frequent than active-voice.

To statistically corroborate these observations, we employed Generalized Estimating Equations (GEE; e.g., Hanley et al., 2003; Hardin and Hilbe, 2003). GEE is an extension of Generalized Linear Models (Nelder and Wedderburn, 1972) that is capable of handling repeated-measures designs as well as mixed designs. Unlike ANOVA—but very much like, e.g., Generalized Linear Mixed Models (GLMMs)—GEE can statistically accommodate a wide range of different data types (categorical frequencies, ordinal data, continuous data, etc.) by providing a variety of appropriate distribution and link functions. However, contrasting with GLMMs, separate by-participant and by-item analyses need to be carried out in GEE, analogous to *F*1 and *F*2 in ANOVA. One advantage of GEE over GLMMs (especially when the latter are used in their recommended “maximal” form, see Barr et al., 2013) is that GEE is computationally less complex and more likely to converge on the type of data we consider here (an issue that becomes more severe with the more complex experimental designs that we introduce later).

Here, the GEE model was used to predict the likelihood of producing a passive-voice (vs. active-voice) paraphrase as a function of Information Status. (Due to rare occurrences of B0, G0, and GB responses, all passive responses were combined into a single category.) Since the dependent variable was binary, a *binomial* distribution and *logit* link function were used (thus implementing a binary logistic regression model). To investigate whether effects generalize across participants and items, two types of binary logistic GEE analyses were carried out: the first took Information Status as a within-participant factor and the second as a within-item factor, each time assuming an exchangeable covariance structure for repeated measurements.

Generalized Score Chi-Squares derived from this analysis showed a clear effect of *Information Status* both by participants [χ^2^_(1)_ = 15.440, *p* < 0.001] and by items [χ^2^_(1)_ = 13.879, *p* < 0.001], whereby passive-voice responses were reliably more likely when the Patient was given (0.54 ± 0.10) (mean ± SE by participants) than when the Agent was given (0.22 ± 0.07).

### Discussion

In Experiment 1, passive-voice responses were overall less frequent than active-voice responses. This is not surprising, since active is the canonical form of transitive description and there was no explicit instruction or other encouragement to utilize the passive-voice.

The givenness manipulation produced a very clear effect, with passive use increasing when the Patient of the event was *given* (i.e., the Patient was introduced earlier in the discourse). This is in agreement with existing subject-assignment literature (see Myachykov et al., 2011): that is, the assignment of a protagonist to subject position drives subsequent structural selection. In this instance, whichever protagonist was given in the stimulus, participants were likely to maintain as the subject in their paraphrase. The most frequent way of topicalizing a protagonist (in English) is via assignment to sentential subject position (others, such as clefting, are much less common; see Keenan and Dryer, 2006). When the subject is the Agent of an action, the canonical active-voice is selected; when the subject is the Patient, passive-voice is selected.

## Experiment two

### Introduction

In Experiment 1 we established a clear effect of information structure, with a given Agent more likely to promote active-voice, and a given Patient more likely to promote passive-voice. As discussed in the introduction, while there is a low degree of agreement in the literature regarding factors that promote get-passive use, suggestions primarily revolve around aspects of the Patient. In light of this relatively unfocussed situation, rather than assigning some highly constrained attribute to the Patient (such as intentionality, control, negative affectedness, etc.), Experiment 2 investigates whether a general focus or emphasis placed on the Patient increases the likelihood of get-passive use. Here, we combined the earlier givenness manipulation with this additional factor.

To establish this general focus or emphasis, we employed a syntactic cleft. This type of “foregrounding construction” (see Keenan and Dryer, 2006) marks one protagonist as being of primary importance in the described event. Significantly, this construction allows either the Agent or Patient to appear in sentence-initial position, without altering the voice or functional assignments within the stimuli; that is, the Patient of an active-voice sentence remains as the Patient, despite appearing in a clefted, sentence-initial position.

If get-passives indeed communicate some form of Patient importance, we should see an increase in the proportion of get-passives when the Patient is focussed via a cleft. This may also interact with the effect of givenness observed in Experiment 1.

### Participants

A new sample of twenty-four native English speakers (age 19–50, mean age 25; 33% males) were tested in individual sessions, with each lasting approximately 30 min. They each received subject payment or course credits for their participation. All participants were recruited through the University of Glasgow's subject database.

### Stimuli

Twenty-four sets of materials were created based on those used in Experiment 1, though in this case, each had four conditional variants (as in 4). Each item was two sentences in length. As before, the second sentence described a transitive event, while the first provided a preamble. The first manipulated factor, *information status*, was established as in Experiment 1.

(4a) *The cowboy rode across the desert into the small dusty town*.
  *It was him who a thief attacked upon arriving*. [given Patient; focussed Patient]
(4b) *The cowboy rode across the desert into the small dusty town*.
  *It was a thief who attacked him upon arriving*. [given Patient; focussed Agent]
(4c) *The thief made her way through the sandstorm to a small town*.
  *It was a cowboy who she attacked upon arriving*. [given Agent; focussed Patient]
(4d) *The thief made her way through the sandstorm to a small town*.
  *It was her who attacked a cowboy upon arriving*. [given Agent; focussed Agent]

In addition to this, the second sentence featured a cleft containing one of the protagonists; that is, either the Agent or Patient of the critical transitive event was foregrounded. This placed a clear focus on that protagonist and constituted the second manipulated factor; focus. The transitive description was always in active-voice; this meant that any changes in passive production would be motivated by the experimental factors. This produced a 2 (Information Status levels) × 2 (Focus levels) design. A full list of materials is given in the appendix (Table A2).

### Procedure

The procedure followed that of Experiment 1. The 24 (items) × 4 (conditions) were assigned to four separate files in such a way that each item appeared precisely once per file, and in a different condition in each of the four files, using a Latin square. This resulted in six items per condition per file, ensuring an equal frequency of each condition in each file.

As before, 50 filler items were also included, giving a total of 74 trials per file; a short practice session was included; and trials proceeded as in Figure 1 above. Responses were coded for voice at three levels (active, be-passive, or get-passive), and passive responses were also coded for by-phrase inclusion.

### Results

In this instance, each of the 24 participants produced at least two syntactic alternatives Again responses that were not transitive were discarded, accounting for less than 1% of the data.

Table 2 shows raw counts of active-voice paraphrases, be-passive paraphrases (with or without an agentive by-phrase), and get-passive paraphrases (with or without a by-phrase) broken down by levels of *Information Status* and *Focus*.

**Table 2:** **C*ross-tabulation results*, giving raw counts of responses (AV: active-voice, B0: be-passive without by-phrase, BB: be-passive with by-phrase, G0: get-passive without by-phrase, GB: get-passive with by-phrase) for the two levels per factor (Information Status and Focus)**.

**Condition**		**Response Type**				
**Given**	**Focus**	**AV**	**B0**	**BB**	**G0**	**GB**
Patient	Patient	53 (0.37)	12 (0.08)	66 (0.46)	4 (0.03)	10 (0.07)
	Agent	59 (0.42)	12 (0.08)	67 (0.47)	3 (0.02)	1 (0.01)
Agent	Patient	95 (0.67)	18 (0.13)	22 (0.15)	5 (0.04)	2 (0.01)
	Agent	106 (0.74)	15 (0.10)	18 (0.13)	1 (0.01)	3 (0.02)
Total		313 (0.55)	57 (0.10)	173 (0.30)	13 (0.02)	16 (0.03)

*Rounded probabilities per condition (respectively per Total) are shown in parentheses*.

Table 2 indicates a similar pattern to Experiment 1, with active-voice responses being dominant, especially following a given Agent; be-passives are also relatively frequent, again overtaking actives following a given Patient. Get-passives are the least frequent form overall, though there is a notable increase in their likelihood over Experiment 1, with most of these appearing in focussed Patient conditions. The inferential analyses below focused on (a) the general likelihood of producing a passive voice paraphrase, (b) the likelihood of producing a be-passive paraphrase, and (c) the likelihood of producing a get-passive paraphrase, respectively.

#### Passive-voice responses

For this stage of analysis, all passive responses (be-passives and get-passives with and without an agentive by-phrase) were combined into a single category. Again, we used binary logistic GEEs (by participants and items, assuming an exchangeable covariance structure for repeated measurements) to model occurrences of passive-voice over active voice paraphrases as a function of *Information Status* and *Focus*, as well as the interaction between them. Table 3 summarizes the results from these analyses.

**Table 3:** **Generalized-Score Chi-Squares from *binary logistic GEE* analyses, predicting the likelihood of passive-voice responses by factor combinations of Information Status (given Agent or given Patient) and Focus (Agent or Patient cleft)**.

**Effect**	**By participants**		**By items**	
	**χ^2^_(1)_**	***P***	**χ^2^_(1)_**	***P***
Information status (I)	12.107	^*^0.001	15.242	^*^0.001
Focus (F)	2.692	0.101	4.164	^*^0.041
I × F	0.061	0.805	0.057	0.812

* *Represents significance level of 0.05*.

There was a main effect of *Information Status*, such that passive-voice paraphrases were reliably more likely when the Patient was given (0.61 ± 0.05) than when the Agent was given (0.29 ± 0.05). There was also a main effect of *Focus* (significant by items only), whereby passive-voice responses were more likely when the Patient was focussed via clefting (0.48 ± 0.03), compared to when the Agent was focussed (0.41 ± 0.04).

#### Be-passive responses

Next, we considered the production of be-passive paraphrases out of all valid responses (active-voice, be-passives, and get-passives). The model was used to predict likelihood of producing a be-passive response as a function of *Information Status* and *Focus*, as well as the interaction between them. Table 4 summarizes these results.

**Table 4:** **Generalized-Score Chi-Squares from *binary logistic GEE* analyses, predicting the likelihood of be-passive responses by factor combinations of Information Status (given Agent or given Patient) and Focus (Agent or Patient cleft)**.

**Effect**	**By participants**		**By items**	
	**χ^2^_(1)_**	***P***	**χ^2^_(1)_**	***P***
Information status (I)	11.487	^*^0.001	13.863	^*^0.001
Focus (F)	0.407	0.524	0.669	0.413
I × F	1.065	0.302	0.658	0.417

* *Represents significance level of 0.05*.

As indicated by Table 4, there was a main effect of *Information Status*, whereby a given Patient reliably increased the likelihood of be-passive paraphrases (0.55 ± 0.06), as compared to a given Agent (0.25 ± 0.04). There was no effect of Focus.

#### Get-passive responses

In this analysis we considered the production of get-passive paraphrases out of all valid responses. The model was used to predict the likelihood of producing a get-passive response as a function of *Information Status* and *Focus*, as well as the interaction between them. Table 5 summarizes these results.

**Table 5:** **Generalized-Score Chi-Squares from *binary logistic GEE* analyses, predicting the likelihood of get-passive responses by factor combinations of Information Status (given Agent or given Patient) and Focus (Agent or Patient cleft)**.

**Effect**	**By participants**		**By items**	
	**χ^2^_(1)_**	***P***	**χ^2^_(1)_**	***P***
Information Status (I)	0.564	0.453	0.441	0.507
Focus (F)	5.046	^*^0.025	3.115	(^*^)0.078
I × F	0.595	0.440	0.190	0.663

* *Represents significance level of 0.05*.

(*) *Denotes approaching significance*.

As indicated by Table 5, there was a main effect of *Focus*, in which the likelihood of get-passive paraphrases was increased when the Patient was focussed via clefting (0.07 ± 0.02), as opposed to when the Agent was focussed (0.03 ± 0.01). This effect was marginal by items, and significant by subjects. There was no effect of *Information Status*.

### Discussion

As in the first experiment, Experiment 2 revealed fewer passive-voice responses overall than active-voice responses. The main effect of Information Status was also maintained from Experiment 1: a given Patient increased the likelihood of passive-voice paraphrases (in complementary distribution with active-voice paraphrases, which were more likely following a given Agent). Importantly, analyses focussing on each passive-type separately indicated that this effect of Information Status mainly affected the likelihood of the more canonical be-passive form rather than the less common get-passive form (as was also suggested in Experiment 1 where get-passive occurrences were very rare).

The additional focus manipulation (via clefting of either Agent or Patient) did not interact with Information Status, but independently influenced responses. Interestingly, in contrast to Information Status, the focus manipulation mainly affected occurrences of get-passive rather than be-passive paraphrases: regardless of which protagonist was *given*, get-passive production was more likely following a story with a clefted Patient in the last sentence rather than a clefted Agent. Given the low probability of get-passive uses in general, it remains to be seen how well this latter finding would replicate in future research. However, it does lend some support to theories which claim that emphasis on the Patient is a significant contributor to the production of get-passives. By contrast, probabilities of be-passives appear to be much less effected by variations in Patient-related focus.

## Experiment three

### Introduction

Experiments 1 and 2 both indicated that, relative to a given Agent, a given Patient reliably promotes the use of passive-voice, and of be-passives in particular. In Experiment 2 we further showed that the use of get-passives, but not the use of be-passives, is promoted by a general focus (or mark of importance) on the Patient of an action via clefting. In Experiment 3 below, we consider the effect of a more specific Patient-related attribute.

As noted earlier, existing literature offers multiple suggestions as to what constitutes the main factor promoting get-passive use; the majority of these suggestions, however, do gravitate toward the event's Patient. Among these diverse proposals, there are several that fall within the attribution of *agentivity* of the Patient, including Patient responsibility or blame, Patient purposefulness, Patient initiative or control, etc. (see Introduction).

Here, we subsume the above into the category of *agentivity*, using Patient-related questions for paraphrasing. In those questions, we either frame the Patient as being more Patient-like (a passive undergoer of an action; e.g., *What happened to the Patient?*) or as more Agent-like (having some active role in the event; e.g., *What did the Patient do?*). These Patient-related questions are also likely to elicit a greater number of passive-voice responses overall (thus increasing the reliability of statistical interpretation).

### Participants

Twenty-four native English speakers (age 18–52, mean age 23; 58% males) were tested in individual sessions, with each lasting approximately 30 min. They each received subject payment or course credits for their participation. All participants were recruited through the University of Glasgow's subject database. These twenty-four participants had not participated in either of the previous experiments.

### Stimuli

Twenty-four sets of materials were created based on those used in Experiment 1. Here, each had four conditional variants (as in 5). Each item was two sentences in length. As in both previous experiments, the second sentence described a transitive event, while the first provided a preamble. The first manipulated factor, *Information Status*, was established as in Experiments 1 and 2.

(5a) *The cowboy rode across the desert into the small dusty town*.
  *When he arrived a thief attacked him*.
  *What happened to the cowboy?* [given Patient; Patient-like]
(5b) *The cowboy rode across the desert into the small dusty town*.
  *When he arrived a thief attacked him*.
  *What did the cowboy do?* [given Patient; Agent-like]
(5c) *The thief made her way through the sandstorm to a small town*.
  *When she arrived she attacked a cowboy*.
  *What happened to the cowboy?* [given Agent; Patient-like]
(5d) *The thief made her way through the sandstorm to a small town*.
  *When she arrived she attacked a cowboy*.
  *What did the cowboy do?* [given Agent; Agent-like]

In addition, rather than having a simple visual prompt to retell the event, participants were presented with one of two types of question, framing the Patient of the action either as being Patient-like (*What happened to the* Patient?) or as being Agent-like (*What did the* Patient*do*?). This constituted the second manipulated factor; Patient Framing. The critical transitive description per story was always in active-voice, ensuring that any changes in passive-voice production would be motivated by the experimental factors. This produced a 2 (Information Status levels) × 2 (Patient Framing levels) design. A full list of materials is given in the appendix (Table A3).

### Procedure

The procedure followed that of Experiment 2, with the 24 (items) × 4 (conditions) being assigned to four separate files in such a way that each item appeared precisely once per file, and in a different condition in each of the four files, using a Latin square. This resulted in six items per condition per file, ensuring an equal frequency of each condition in each file.

As in both previous experiments, 50 filler items were also included, giving a total of 74 trials per file; a short practice session was included in the format of the main experiment; and trials once again proceeded as in Figure 1 above. Responses were coded for voice at three levels (active, be-passive, or get-passive), and passive responses were also coded for by-phrase inclusion.

### Results

As stated throughout, we were concerned with the production of syntactic alternatives, and deemed it necessary for participants to demonstrate the availability of at least two syntactic forms. In this instance, all participants produced at least two alternatives, and were therefore included in the subsequent analyses. Less than 0.5% of the responses were non-transitive and therefore excluded from analysis. Table 6 shows raw counts for the five response types (active-voice, be-passive with or without an agentive by-phrase, get-passive with or without a by-phrase) broken down by *Information Status* (Patient given or Agent given) and *Patient Framing* (Patient-like or Agent-like).

**Table 6:** **C*ross-tabulation results*, giving raw counts of responses (AV, active-voice; B0, be-passive without by-phrase; BB, be-passive with by-phrase; G0, get-passive without by-phrase; GB, get-passive with by-phrase) for the two levels per factor (Information Status and Patient Framing)**.

**Condition**		**Response type**				
**Given**	**Framing**	**AV**	**B0**	**BB**	**G0**	**GB**
Patient	Patient-like	12 (0.07)	24 (0.14)	87 (0.52)	18 (0.11)	25 (0.15)
	Agent-like	31 (0.18)	23 (0.14)	70 (0.42)	20 (0.12)	24 (0.14)
Agent	Patient-like	4 (0.02)	34 (0.20)	96 (0.57)	11 (0.07)	23 (0.14)
	Agent-like	16 (0.10)	25 (0.15)	92 (0.55)	14 (0.08)	20 (0.12)
Total		63 (0.09)	106 (0.16)	345 (0.52)	63 (0.09)	92 (0.14)

*Rounded probabilities per condition (respectively per Total) are shown in parentheses*.

Table 6 indicates a markedly different distributional pattern compared to that in Experiments 1 and 2. Active-voice responses are no longer dominant, and most actives now appear following a given *Patient* (as opposed to a given Agent in the first two experiments). Be-passives now take over as by-far the most frequent response type; contrary to both Experiments 1 and 2, most be-passives appear following a given *Agent*, rather than a given Patient as previously seen. Get-passives are the least frequent form overall, though there is a notable increase in their absolute frequency over Experiments 1 and 2, with most get-passives appearing in given Patient conditions (the conditions in which *be-passives* were previously observed to be more likely).

#### Passive-voice responses

As before, data were analyzed using binary logistic GEEs (by participants and items) treating *Information Status* and *Patient Framing* as repeated-measures predictors with exchangeable covariance structure. In the first analysis, all passive responses (be-passives and get-passives with and without a by-phrase) were combined into a single category and the models were set up to predict occurrences of passive-voice paraphrases as a function of *Information Status* and *Patient Framing*, as well as the interaction between them. Table 7 shows the results.

**Table 7:** **Generalized-Score Chi-Squares from *binary logistic GEE* analyses, predicting the likelihood of passive-voice responses by factor combinations of Information Status (given Agent or given Patient) and Patient Framing (Agent-like or Patient-like)**.

**Effect**	**By participants**		**By items**	
	**χ^2^_(1)_**	***P***	**χ^2^_(1)_**	***P***
Information status (I)	3.989	^*^0.046	6.469	^*^0.011
Patient framing (PF)	8.730	^*^0.003	9.622	^*^0.002
I × PF	0.484	0.487	0.278	0.598

* *Represents significance level of 0.05*.

As indicated by Table 7, there was a main effect of *Information Status*, which was in the opposite direction to that seen in both Experiments 1 and 2: passive-voice paraphrases were now more likely when the Agent was given (0.95 ± 0.03), as opposed to when the Patient was given (0.88 ± 0.05). There was also a main effect of *Patient Framing*, whereby passive-voice responses were more likely when the questions framed the Patient as more Patient-like (0.96 ± 0.03), compared to when the questions framed the Patient as more Agent-like (0.87 ± 0.05).

### Be-passive responses

Next, we considered the production of be-passive paraphrases out of all valid responses, i.e., the binary logistic GEE models were used to predict likelihood of producing a be-passive response as a function of *Information Status* and *Patient Framing*, as well as the interaction between them. Table 8 summarizes these results.

**Table 8:** **Generalized-Score Chi-Squares from *binary logistic GEE* analyses, predicting the likelihood of be-passive responses by factor combinations of Information Status (given Agent or given Patient) and Patient Framing (Agent-like or Patient-like)**.

**Effect**	**By participants**		**By items**	
	**χ^2^_(1)_**	***P***	***GS*_χ^2^_**	***P***
Information status (I)	11.191	^*^0.001	9.230	^*^0.002
Patient framing (PF)	4.137	^*^0.042	5.724	^*^0.017
I × PF	0.223	0.637	0.120	0.729

* *Represents significance level of 0.05*.

As indicated, there was a main effect of *Information Status*, again the inverse of that seen in the previous experiments: a given Agent reliably increased the likelihood of a be-passive paraphrase (0.74 ± 0.11), as compared to a given Patient (0.61 ± 0.12). There was also a main effect of *Patient Framing*, whereby be-passives were significantly more frequent after a question framing the Patient as Patient-like (0.72 ± 0.12) than after a question framing the Patient as Agent-like (0.63 ± 0.12). The interaction did not approach significance.

#### Get-passive responses

In this analysis we considered the production of get-passives out of all valid responses. The GEE model was used to predict likelihood of producing a get-passive response as a function of *Information Status* and *Patient Framing*, as well as the interaction between them. Table 9 summarizes these results.

**Table 9:** **Generalized-Score Chi-Squares from *binary logistic GEE* analyses, predicting the likelihood of get-passive responses by factor combinations of Information Status (given Agent or given Patient) and Patient Framing (Agent-like or Patient-like)**.

**Effect**	**By participants**		**By items**	
	**χ^2^_(1)_**	***P***	**χ^2^_(1)_**	***P***
Information status (I)	3.380	^(^*^)^0.066	5.912	^*^0.015
Patient Framing (PF)	0.003	0.958	0.004	0.951
I × PF	0.003	0.960	0.001	0.980

* *Represents significance level of 0.05*.

(*) *Denotes approaching significance*.

As shown, there was a main effect of *Information Status*, which was absent from the data in Experiment 2: get-passive paraphrases were reliably more likely after a given Patient (0.26 ± 0.11) than after a given Agent (0.20 ± 0.10). This effect was only marginal by subjects, but significant by items. There was no effect of *Framing*.

### By-phrase inclusion in passive responses

In Experiments 1 and 2, we have not reported analyses of by-phrase inclusion, since the relevant descriptive statistics indicated that it was rather uncommon for participants to drop the by-phrase, and hence inferential statistics could not be informatively applied. However, Experiment 3 elicited a far greater number of passives overall, allowing a more informative consideration of by-phrase inclusion.

The binary logistic GEE models were used to predict the likelihood of including an agentive by-phrase in a passive response as a function of *Information Status, Patient Framing*, and *Passive-type* (whether participants used *be* or *get* to form their passive response), and all possible interactions between these factors. Results are summarized in Table 10.

**Table 10:** **Generalized-Score Chi-Squares from *binary logistic GEE* analyses, predicting the likelihood of agentive by-phrase inclusion by factor combinations of Information Status (given Agent or given Patient), Patient Framing (Agent-like or Patient-like), and Passive-type (be or get)**.

**Effect**	**By participants**		**By items**	
	**χ^2^_(1)_**	***P***	**χ^2^_(1)_**	***P***
Information Status (I)	0.312	0.577	0.601	0.438
Patient Framing (PF)	0.424	0.515	0.167	0.683
Passive Type (PT)	4.750	^*^0.029	7.948	^*^0.005
I × PF	0.562	0.453	0.220	0.639
I × PT	0.480	0.488	0.905	0.342
PF × PT	0.146	0.702	0.162	0.687
I × PF × PT	0.315	0.574	0.258	0.612

* *Represents significance level of 0.05*.

As is evident, only one significant effect was established in this analysis, namely a main effect of *Passive-type*, whereby by-inclusion was reliably increased when the passive-type used was *be* (0.75 ± 0.08) rather than *get* (0.61 ± 0.14).

### Discussion

The main effect of Information Status that was observed in the first two experiments is entirely reversed in Experiment 3. That is, a given Agent now increased the likelihood of passive-voice (rather than active-voice), while a given Patient increased the likelihood of active-voice responses. Note that this effect is mainly driven by be-passive responses (which now constitute the majority of responses overall), whereas the effect of Information Status on get-passives is smaller, and in the opposite direction, therefore comparable to the effect of Information Status on *be-passives* in Experiments 1 and 2.

The latter is interesting since Information Status previously had no effect on get-passive likelihood, but now it displays a main effect such that get-passive paraphrases become more likely when the Patient is given (comparable to the effect of Information Status previously observed for be-passive uses). In other words, the effect of Information Status on be-passive uses is now reversed, yet the original effect appears to have transferred to get-passive uses, as if *get* is now filling the role previously filled by *be*.

Clearly, the Patient-related questions in Experiment 3 (as opposed to free paraphrasing in Experiments 1 and 2) must be at least partly responsible for these changes in the effects of Information Status. Consider that the Patient-related questions in Experiment 3 actually introduced additional information to the discourse established by the story. While the story establishes which of the two protagonists (Agent or Patient) is given vs. new, the Patient-related question provides a highly effective cue to topicality; that is, the question indicates that the Patient is the topic of the on-going discourse, cueing its use as the sentential subject in the response (as a result of which, the overall number of passive-voice responses goes up). This in turn means that the Information Status manipulation in the story no longer acts as a cue for subject-assignment, and hence passive vs. active selection.

Further evidence for the claim that the Patient-related questions contribute to the information status of the Patient comes from the use of pronouns (as opposed to full noun phrases) to refer to the Patient protagonist in the story. A re-inspection of the responses produced in Experiments 1 and 2 (free paraphrasing) revealed very infrequent uses of Patient-referring pronouns (~5%), whereas in Experiment 3, participants very frequently (>80%) started their answer to the Patient-related question with a pronoun.

The short stories and the Patient-related questions each contribute to the overall discourse, with each of these contributions having their own *topic*. The first contribution in the discourse is the same as in Experiments 1 and 2: Information Status (givenness) introduces one protagonist before the other, driving the interpretation of the earlier one as the *topic* of the story. Here, the second contribution to the discourse comes in the form of the question/answer interaction of the task (contrasting with the first two experiments where participants simply had to re-tell the story in their own words): participants are always prompted with a Patient-related question, establishing the Patient as the *topic* of the task interaction between participant and experiment.

Since the question is always Patient-related, the given protagonist of the task interaction is necessarily the Patient. The given protagonist of the first contribution can be the Agent or the Patient (as established via the story). This gives two possible situations: in the first, the givenness of the Agent and Patient is somewhat balanced, with part of the discourse marking the *Agent* as given (in the story) and another part marking the *Patient* as given (via the experimental task). In the second instance, the Patient is consistently given in the discourse. Be-passives are more frequent in the former (balanced) situation as compared to the latter (“Patient-heavy”) situation. This is in line with the finding that be-passives show no preference for one protagonist over the other and tend to report the whole event, including the Agentive by-phrase. In the latter (unbalanced, “Patient-heavy”) situation, the likelihood of get-passive use increases. This is also in line with the findings of Experiment 2, which indicated that get-passives occur more frequently when the Patient is marked as important.

It appears that get-passives still display an affinity for the Patient. When the Patient is both given and target of the question, it is unambiguously marked as the more important protagonist; when the Agent is given, yet the Patient is the target of the question, the attention, or importance is distributed between the two protagonists. In the former situation, with an important Patient, get-passives responses are more likely than when the Patient is not marked as important.

When the Patient is framed as Patient-like (i.e., as having passive involvement in the event), the probability of be-passive use increases. This may reflect the interpretation that the Patient was not actively involved in the event in terms of control, responsibility, or blame (all aspects of agentivity; see section Experiment Three: Introduction and our general Introduction). Notably, there was no effect of Patient framing on get-passive likelihood. While this does not directly contradict the suggestion that get-passives indicate Patient agentivity, it indicates that be-passives are preferred in the *absence of an agentive Patient*, rather than get-passives being preferred in the presence of one.

Finally, in agreement with recent corpus findings, the inclusion of an agentive by-phrase was significantly more likely in conjunction with a be-passive, rather than a get-passive response. This is further evidence that the be-passive implies more of an equal status between Agent and Patient, respectively, that the get-passive focusses more strongly on the Patient, to the extent that the Agent is significantly more likely to be completely dropped in a get-passive sentence.

## General discussion

The current literature on passive uses in English is broad but experimentally weak. While suggestions abound with regard to the semantic and syntactic differences between get- and be-passives, there is very little agreement on specifics. Our aim in this paper has been to provide experimental evidence in an area dominated by theory.

Here we concentrated on *information structure* and attributes of the *passive Patient*. While the former has previously been shown to affect voice selection, its impact on the choice of get-passive vs. be-passive required exploration. With regard to the latter, the passive Patient is frequently cited as having some manner of heightened significance in the get-passive; we considered two aspects that recur in the literature: a general importance assigned to the Patient, and a sense of Patient agency.

Our focus was on how these factors influence the use of active-voice vs. passive-voice, as well as be-passives vs. get-passives. Employing a paraphrasing paradigm, we manipulated aspects of information structure and focus. This task allowed a natural way to determine the order in which protagonists were introduced and to apply additional focus via clefting or question formations.

Information Status (given Agent or given Patient) was consistently manipulated across all three experiments. Experiment 1 used only this manipulation, while the subsequent experiments additionally considered aspects of the passive Patient: focus/importance (Experiment 2), and Patient agency (Experiment 3).

In the overall pattern of results, the primary driver of differences in the response data is *givenness*, established in each experiment via the order that the Agent and Patient appear in the story. In Experiments 1 and 2, a given Agent promotes active-voice use, and a given Patient promotes passive-voice. In Experiment 3, the reverse is true (discussed further below).

Givenness clearly had the biggest effect in purely quantitative terms. However, when considering the pattern of all significant effects, regardless of the shift in absolute numbers, a more interesting picture emerges. We see that each alternative form (active, be-passive, get-passive) responds uniquely to the various manipulations. Rather than serving to modulate the strong effect of givenness, we observe distinct main effects, with no interactions between the various manipulations.

Experiment 1 revealed a clear effect of Information Status, whereby a given Agent resulted in more *active-voice* responses, while a given Patient resulted in more *be-passive* responses. This effect was maintained in Experiment 2, where we also found that relative get-passive frequency increased when the Patient was marked as important via clefting. Experiment 3 suggested that be-passives were more common when the Agent and Patient were balanced in terms of importance, while get-passives were again more common when the Patient was marked as important.

Taking the findings of these experiments together, it is not the case that the be-passive and get-passive combine simply into a single category; rather we can see that no one manipulation affects get-passives and be-passives in the same way, and each of the three transitive descriptions responds to a separate set of manipulations.

The high overall frequency of be-passives creates the appearance that active-voice and passive-voice are in complementary distribution; however, looking more closely, it is active-voice vs. *be-passive* in particular that occupy a near-complementary relationship. Their selection is driven by which of the protagonists is established as given, the Agent or the Patient. A given Agent results in more active-voice responses, while a given Patient gives rise to more be-passive responses, as was the case in Experiments 1 and 2. As for *get* and *be*, while both are passive forms, it is only get-passives that increase in likelihood when the Patient is marked as important or is in focus; this is regardless of whether or not the Patient is the given protagonist in the paraphrased story. Get-passives do not seem to respond to the factors that drive the selection of both active-voice and be-passive.

Since Experiments 1 and 2 each had only one aspect contributing to the discourse (i.e., *givenness*, as established via order of mention in the story), there was only this one aspect that participants could utilize to inform their syntactic choices. This resulted in a clear effect of Information Status whereby a given Patient prompted more passive-voice (Patient-first) responses, and a given Agent prompted more active-voice (Agent-first) responses. However, in Experiment 3, there were two aspects contributing to the discourse. The first was information structure within the story, as in the earlier experiments. The second was introduced by the Patient-related question as part of the experimental task. This secondary aspect, in directing a question at the Patient, marked the Patient as the on-going discourse topic. This overrides the information structure manipulation of the stories, making the Patient consistently *given* (in the sense of *the given topic as per the question*). This is clearly reflected in the high overall proportion of passive-voice responses (and the frequent use of Patient-referring pronouns) in Experiment 3 as compared with Experiments 1 and 2.

Having established that Experiment 3 involves two contributions to the discourse, this gives rise to implications for selection of passive-type. When there is a balance (Agent and Patient are each marked as given: one by the story; one by the question), be-passives are preferred; when there is a heavy weighting toward the Patient (Patient is marked as given in both), get-passive use increases. This supports the above suggestion that get-passives respond to a Patient that is marked as important, while be-passives give a more balanced status to Agent and Patient. This is supported also by the finding that agentive by-phrases are more likely with *be* than with *get*; that is, the whole event tends to be mentioned in the be-passive, while the Patient is given higher priority in the get-passive, to the likely exclusion of the Agent.

It is notable that in Experiments 1 and 2, the likelihood of using a personal pronoun (*he*, *she*, or *they*) in reference to the Patient was less than 5%, while in Experiment 3 it is greater than 80%. This clearly supports the suggestion that there are two contributors to the discourse in the final experiment, with the latter (question) overriding the givenness of the former (the story): the Patient becomes so clearly *given* or established via the question that it no longer requires explicit specification and can be reduced to a pronoun. For Gundel (1988, 1999), this is part of referential givenness; the target entity is *referentially given*, and using a full noun-phrase (such as *“the cowboy”*) would be over-specific.

If be-passives and get-passives were functionally or semantically equivalent, we would expect to see both passives forms increasing in frequency in response to a factor such as givenness. Our data do not support such a view. While passives do increase overall following a given Patient, this is driven by the high frequency of be-passive responses: when considering each passive-type separately during free paraphrasing (Experiments 1 and 2), it is clear that be-passive responses increase following a given Patient, while get-passives are largely unaffected; on the other hand, the use of get-passives, but not the use of be-passives, is affected by focussing the Patient via clefting (Experiment 2). Furthermore, there are no interactions within the three experiments; rather there are a series of main effects, with each of the three forms (active, be-passive, get-passive) being selectively sensitive to one or the other experimental manipulation. In Experiment 3 (where participants had to answer Patient-related questions), we even find a situation whereby a single experimental manipulation (information status) produces *opposing* trends with respect to get- vs. be-passive usage.

The findings discussed here suggest that these three transitive descriptions (active, be-passive, and get-passive) are distinct to some extent. While they vary in terms of frequency, both in our data and in corpora (active-voice > be-passive > get-passive), and their use overlaps, each has distinct motivating factors.

We can conceptualize these differences as variations along two dimensions: (1) focus on or importance of the Patient within an event (*Patient Importance*), and (2) the functional prominence of the Patient as indicated by assignment to a prominent syntactic role, e.g., subject (*Patient Prominence*). These dimensions and their application to each transitive description are given in Table 11.

**Table 11:**
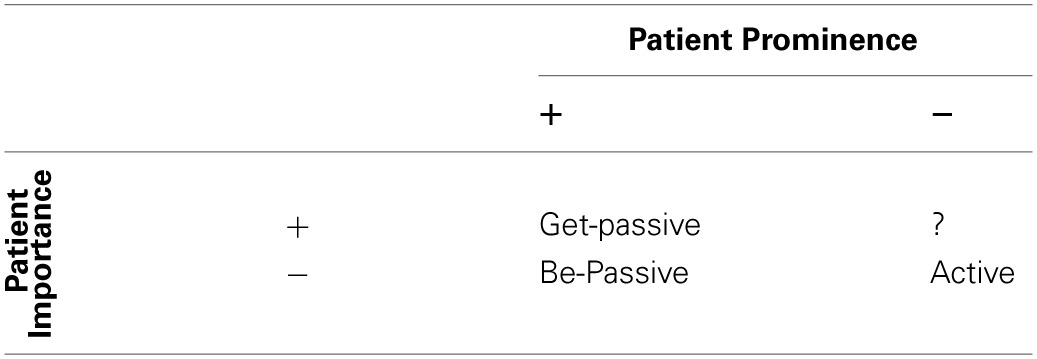
**Distribution of the three types of transitive description (active-voice, be-passive, get-passive) along two dimensions (Patient Importance and Patient Prominence)**.

Active-voice and (specifically) be-passive descriptions have in common a lack of investment in the Patient (−[Patient Importance]), but are separated by the fact that active is used when, due to givenness, the Agent is assigned to subject position (−[Patient Prominence]), and the be-passive is used when the Patient is assigned to subject position (+[Patient Prominence]).

On the other hand, the common factor between be-passives and get-passives is their Patient-subject (+[Patient Prominence]), while they are separated by the fact that the get-passive makes stronger assumptions or implications about the Patient. Specifically, the get-passive implies that the Patient is more important, or at least that it has the greater focus in the event (+[Patient Importance]), while the be-passive makes no such implication (−[Patient Importance]).

In this paper we have concentrated on three constructions (active-voice, be-passive, and get-passive), none of which can fill the role of +[Patient Importance], −[Patient Prominence], indicated by the “?” in Table 11. A possible candidate for this role is the clefting construction utilized in Experiment 2, however, the use of a syntactic cleft is not restricted to active-voice, and therefore not restricted to having the -[Patient Prominence] feature. This deserves further exploration in future research.

It is generally assumed that the most significant distinction in transitive descriptions is that of active-voice vs. passive-voice, while the *get* vs. *be* distinction is a rather minor matter within the latter of these voice options; this is perhaps because there is a more obvious structural difference between active and passive. Certainly, the [Patient Prominence] dimension discussed above is the strongest driver of difference. However, in terms of usage and functional attributes, it would appear that there is as much qualitative difference between active-voice and be-passive (along the [Patient Prominence] dimension), as there is between be-passive and get-passive (along the [Patient Importance] dimension). Each of the three options has its own unique combination of features, providing three distinct options in transitive event description.

### Conflict of interest statement

The authors declare that the research was conducted in the absence of any commercial or financial relationships that could be construed as a potential conflict of interest.

## Footnotes

^1^Throughout this paper, we will use the labels *Agent* and *Patient* as shorthand for the more appropriate “generalized role” terms *Proto-Agent* and *Proto-Patient* (e.g., Dowty, 1991), or *Actor* and *Undergoer* (e.g., Foley and Van Valin, 1984; Van Valin and LaPolla, 1997), respectively.

^2^The subject pool includes over 6000 registered users from various departments across the university between the ages of 16 and 90. Participants who meet the relevant criteria receive an email giving details of the study, and are signed up on a first-come, first-served basis. Participants across the three experiments reported here had a mean age of 24; the majority were undergraduate students of University of Glasgow, and all had at least a high school education.

## Appendix

**Table A1:** **Experiment one**.

**Item**	**Condition**	**Story**
1	1	The actor came to the end of a long day on the set. As he walked across to his trailer an officer arrested him.
1	2	The officer began his shift late in the day. In the course of his long evening he arrested an actor.
2	1	The tramp searched the streets for a quiet place to weather the night. As he lay in an alley, a youth kicked him.
2	2	The youth wandered the streets looking to cause trouble. At the end of a quiet alley, he kicked a tramp.
3	1	The cowboy rode across the desert into the small dusty town. When he arrived a thief attacked him.
3	2	The thief made her way through the sandstorm to a small town. When she arrived she attacked a cowboy.
4	1	The scientist spent the day categorizing all the new finds her team had made. When she thought she had finished, an assistant called her.
4	2	The assistant spend her day working on a the new finds from that week. She needed to check something so she called a scientist.
5	1	The courier carried important packages across the wasteland, sometimes exposing him to danger. On his latest trip a hunter caught him.
5	2	The hunter scoured the wastes for unsuspecting caravans and abandoned homes. During a recent trip he caught a courier.
6	1	The soldier served rather unwillingly in a multi-national corporation. As a result the manager fired him.
6	2	The manager ran his far-reaching company very strictly. Just the other day he fired an assistant.
7	1	The chemist enjoyed the scenery as he walked home from work. As he crossed a quiet road a motorist suddenly hit him.
7	2	The motorist drove along some of the quieter roads in the town. As he turned up the main street he suddenly hit a chemist.
8	1	The spy crawled through the vent into the heart of the dam complex. Just as he reached the control room a guard killed him.
8	2	The guard prowled the metal hallways of the secret structure below the dam. As he entered the bottling room he killed a spy.
9	1	The author had recently finalized a manuscript for submission. After some formal checks the published paid him.
9	2	The publisher had commissioned a new piece for his autumn releases. After receiving the manuscript he paid the author.
10	1	The lawyer worked many difficult cases in the big commercial city. Just a few weeks ago, a killer hired him.
10	2	The killer knew he was in trouble and needed to do something about it quickly. After some research, he hired a lawyer.
11	1	The ninja could sneak through any room undetected, hiding in the shadows. Emerging through a doorway a sniper shot him.
11	2	The sniper lay silently on the rooftop, unmoved by the harsh weather. Peering then through his sights he shot a ninja.
12	1	The prisoner had remained in jail for many long years. At last his parole came through and the warden released him.
12	2	The warden had run this jail for decades and had overseen many changes. Today a parole came through and he released a prisoner.
13	1	The waiter had not worked at the restaurant for long, but suspicions had already arisen. Finally today the sheriff detained him.
14	1	The runner had many menial tasks and duties backstage at the concert. Today after several technical problems the singer pushed him.
14	2	The singer became angry after technical problems had delayed their performance. As he stormed around backstage he pushed a runner.
15	1	The juror listened to a very difficult case which took weeks to conclude. In the closing days of the trial the defendant punched him.
15	2	The defendant sat emotionless through most of his lengthy trial. As the conclusion drew near he punched a juror.
16	1	The nurse spent her day off at home catching up on the TV shows she had missed. Right in the middle of a gripping episode the surgeon rang her.
16	2	The surgeon knew it would be a long shift as news of a terrible accident reached him. Extra staff were needed, so in between operations he rang a nurse.
17	1	The burglar hid the stolen items and tried to blend in with the other people in the street. After walking along a couple of streets a policeman trapped him.
17	2	The policeman heard that a robbery had taken place a few streets away. As he moved toward the area he trapped a robber.
18	1	The reporter tried desperately to hunt down a good story but without success. With such a poor performance the editor sacked him.
18	2	The editor enforced strict deadlines and demanded quality stories from all staff. After failing to meet the standard he sacked a reporter.
19	1	The pupil disliked the lessons so she talked and laughed loudly. She caused disruption in the classroom and the teacher slapped her.
19	2	The teacher hoped to inform the unruly class about history and geography. Although she tried to stay calm she slapped a pupil.
20	1	The doctor had worked at the mental asylum for many years and was well known. During a routine checkup a patient murdered him.
20	2	The patient had resided at the mental hospital for many long years. During a normal monthly checkup he murdered a doctor.
21	1	The photographer had managed to capture some clear and compromising pictures. To avoid publication the celebrity compensated him.
21	2	The celebrity liked to control the pictures and information that magazines printed about her. To avoid a scandal, yesterday she compensated a photographer.
22	1	The secretary had attended interviews at several businesses in the financial district. It was good news for her salary when a director appointed her.
22	2	The director found it very difficult to keep with his work when he had so much admin to attend to. Eventually he appointed a secretary.
23	1	The hiker spent his day moving up the mountain toward base camp. As he moved through an area of dense trees a hunter stabbed him.
23	2	The hunter had spent ages in the forest and began to get disorientated. In an automatic response to movement, he stabbed a hiker.
24	1	The captive endured a long time not knowing what would happen to him. He guessed a ransom had arrived when the kidnapper freed him.
24	2	The kidnapper had held himself together and made his demands. Though surprised that the random arrived, he freed the captive.

*Conditions: 1, Patient given; 2, Agent given*.

**Table A2:** **Experiment two**.

**Item**	**Condition**	**Story**
1	1	The actor came to the end of a long day on the set. It was him who an officer arrested that day.
1	2	The officer began his shift late in the day. It was an actor who he arrested that day.
1	3	The actor came to the end of a long day on the set. It was an officer who arrested him that day.
1	4	The officer began his shift late in the day. It was him who arrested an actor that day.
2	1	The tramp searched the streets for a quiet place to weather the night. It was him who a youth kicked in an alley.
2	2	The youth wandered the streets looking to cause trouble. It was a tramp who he kicked in an alley.
2	3	The tramp searched the streets for a quiet place to weather the night. It was a youth who kicked him in an alley.
2	4	The youth wandered the streets looking to cause trouble. It was him who kicked a tramp in an alley.
3	1	The cowboy rode across the desert into the small dusty town. It was him who a thief attacked upon arriving.
3	2	The thief made her way through the sandstorm to a small town. It was a cowboy who she attacked upon arriving.
3	3	The cowboy rode across the desert into the small dusty town. It was a thief who attacked him upon arriving.
3	4	The thief made her way through the sandstorm to a small town. It was her who attacked a cowboy upon arriving.
4	1	The scientist spent the day categorizing all the new finds her team had made. It was her who an assistant called for information.
4	2	The assistant spend her day working on a the new finds from that week. It was a scientist who she called for information.
4	3	The scientist spent the day categorizing all the new finds her team had made. It was an assistant who called her for information.
4	4	The assistant spend her day working on a the new finds from that week. It was her who called a scientist for information.
5	1	The courier carried important packages across the wasteland, sometimes exposing him to danger. It was him who a hunter caught during a recent trip.
5	2	The hunter scoured the wastes for unsuspecting caravans and abandoned homes. It was a courier who he caught during a recent trip.
5	3	The courier carried important packages across the wasteland, sometimes exposing him to danger. It was a hunter who caught him during a recent trip.
5	4	The hunter scoured the wastes for unsuspecting caravans and abandoned homes. It was him who caught a courier during a recent trip.
6	1	The soldier served rather unwillingly in a multi-national corporation. It was him who the manager fired just the other day.
6	2	The manager ran his far-reaching company very strictly. It was a soldier who he fired just the other day.
6	3	The soldier served rather unwillingly in a multi-national corporation. It was the manager who fired him just the other day.
6	4	The manager ran his far-reaching company very strictly. It was him who fired a solider just the other day.
7	1	The chemist enjoyed the scenery as he walked home from work. It was him who a motorist suddenly hit on a quiet road.
7	2	The motorist drove along some of the quieter roads in the town. It was a chemist who he suddenly hit on a quiet road.
7	3	The chemist enjoyed the scenery as he walked home from work. It was a motorist who suddenly hit him on a quiet road.
7	4	The motorist drove along some of the quieter roads in the town. It was him who suddenly hit a chemist on a quiet road.
8	1	The spy crawled through the vent into the heart of the dam complex. It was him who a guard killed at the entrance of the bottling room.
8	2	The guard prowled the metal hallways of the secret structure below the dam. It was a spy who he killed at the entrance of the bottling room.
8	3	The spy crawled through the vent into the heart of the dam complex. It was a guard who killed him at the entrance of the bottling room.
8	4	The guard prowled the metal hallways of the secret structure below the dam. It was him who killed a spy at the entrance of the bottling room.
9	1	The author had recently finalized a manuscript for submission. It was him who the publisher paid after some formal checks.
9	2	The publisher had commissioned a new piece for his autumn releases. It was the author who he paid after some formal checks.
9	3	The author had recently finalized a manuscript for submission. It was the publisher who paid him after some formal checks.
9	4	The publisher had commissioned a new piece for his autumn releases. It was him who paid the author after some formal checks.
10	1	The lawyer worked many difficult cases in the big commercial city. It was him who a killer hired a few weeks ago.
10	2	The killer knew he was in trouble and needed to do something about it quickly. It was a lawyer who he hired a few weeks ago.
10	3	The lawyer worked many difficult cases in the big commercial city. It was a killer who hired him a few weeks ago.
10	4	The killer knew he was in trouble and needed to do something about it quickly. It was him who hired a lawyer a few weeks ago.
11	1	The ninja could sneak through any room undetected, hiding in the shadows. It was him who a sniper shot while emerging from a doorway.
11	2	The sniper lay silently on the rooftop, unmoved by the harsh weather. It was a ninja who he shot while emerging from a doorway.
11	3	The ninja could sneak through any room undetected, hiding in the shadows. It was a sniper who shot him while emerging from a doorway.
11	4	The sniper lay silently on the rooftop, unmoved by the harsh weather. It was him who shot a ninja while emerging from a doorway.
12	1	The prisoner had remained in jail for many long years. It was him who the warden released after the recent parole hearing.
12	2	The warden had run this jail for decades and had overseen many changes. It was the prisoner who he released after the recent parole hearing.
12	3	The prisoner had remained in jail for many long years. It was the warden who released him after the recent parole hearing.
12	4	The warden had run this jail for decades and had overseen many changes. It was him who released a prisoner after the recent parole hearing.
13	1	The waiter had not worked at the restaurant for long, but suspicions had already arisen. It was him who the sheriff detained today.
13	2	The sheriff had almost finished a very long day, but had one last stop to make. It was a waiter who he detained today.
13	3	The waiter had not worked at the restaurant for long, but suspicions had already arisen. It was the sheriff who detained him today.
13	4	The sheriff had almost finished a very long day, but had one last stop to make. It was him who detained a waiter today.
14	1	The runner had many menial tasks and duties backstage at the concert. It was him who the singer pushed after a frustrating day.
14	2	The singer became angry after technical problems had delayed their performance. It was a runner who he pushed after a frustrating day.
14	3	The runner had many menial tasks and duties backstage at the concert. It was the singer who pushed him after a frustrating day.
14	4	The singer became angry after technical problems had delayed their performance. It was him who pushed a runner after a frustrating day.
15	1	The juror listened to a very difficult case which took weeks to conclude. It was him who the defendant punched in the closing days.
15	2	The defendant sat emotionless through most of his lengthy trial. It was a juror who he punched in the closing days.
15	3	The juror listened to a very difficult case which took weeks to conclude. It was the defendant who punched him in the closing days.
15	4	The defendant sat emotionless through most of his lengthy trial. It was him who punched a juror in the closing days.
16	1	The nurse spent her day off at home catching up on the TV shows she had missed. It was her who the surgeon rang urgently.
16	2	The surgeon knew it would be a long shift as news of a terrible accident reached him. It was the nurse who he rang urgently.
16	3	The nurse spent her day off at home catching up on the TV shows she had missed. It was the surgeon who rang her urgently.
16	4	The surgeon knew it would be a long shift as news of a terrible accident reached him. It was him who rang a nurse urgently.
17	1	The burglar hid the stolen items and tried to blend in with the other people in the street. It was him who a policeman trapped in an alley.
17	2	The policeman heard that a robbery had taken place a few streets away. It was a robber who he trapped in an alley.
17	3	The burglar hid the stolen items and tried to blend in with the other people in the street. It was a policeman who trapped him in an alley.
17	4	The policeman heard that a robbery had taken place a few streets away. It was him who trapped a robber in an alley.
18	1	The reporter tried desperately to hunt down a good story but without success. It was him who the editor sacked for poor performance.
18	2	The editor enforced strict deadlines and demanded quality stories from all staff. It was a reporter who he sacked for poor performance.
18	3	The reporter tried desperately to hunt down a good story but without success. It was the editor who sacked him for poor performance.
18	4	The editor enforced strict deadlines and demanded quality stories from all staff. It was him who sacked a reporter for poor performance.
19	1	The pupil disliked the lessons so she talked and laughed loudly. It was her who the teacher slapped for causing disruption.
19	2	The teacher hoped to inform the unruly class about history and geography. It was a pupil who she slapped for causing disruption.
19	3	The pupil disliked the lessons so she talked and laughed loudly. It was the teacher who slapped her for causing disruption.
19	4	The teacher hoped to inform the unruly class about history and geography. It was her who slapped a pupil for causing disruption.
20	1	The doctor had worked at the mental asylum for many years and was well known. It was him who a patient murdered during a routine checkup.
20	2	The patient had resided at the mental hospital for many long years. It was a doctor who he murdered during a routine checkup.
20	3	The doctor had worked at the mental asylum for many years and was well known. It was a patient who murdered him during a routine checkup.
20	4	The patient had resided at the mental hospital for many long years. It was him who murdered a doctor during a routine checkup.
21	1	The photographer had managed to capture some clear and compromising pictures. It was him who the celebrity compensated him to avoid publication.
21	2	The celebrity liked to control the pictures and information that magazines printed about her. It was a photographer who she compensated to avoid publication.
21	3	The photographer had managed to capture some clear and compromising pictures. It was the celebrity who compensated him to avoid publication.
21	4	The celebrity liked to control the pictures and information that magazines printed about her. It was her who compensated a photographer to avoid publication.
22	1	The secretary had attended interviews at several businesses in the financial district. It was her who a director appointed with a pleasing salary.
22	2	The director found it very difficult to keep with his work when he had so much admin to attend to. It was a secretary who he appointed with a pleasing salary.
22	3	The secretary had attended interviews at several businesses in the financial district. It was a director who appointed her with a pleasing salary.
22	4	The director found it very difficult to keep with his work when he had so much admin to attend to. It was him who appointed a secretary with a pleasing salary.
23	1	The hiker spent his day moving up the mountain toward base camp. It was him who a hunter stabbed among the dense trees.
23	2	The hunter had spent ages in the forest and began to get disorientated. It was a hiker who he stabbed among the dense trees.
23	3	The hiker spent his day moving up the mountain toward base camp. It was a hunter who stabbed him among the dense trees.
23	4	The hunter had spent ages in the forest and began to get disorientated. It was him who stabbed a hiker among the dense trees.
24	1	The captive endured a long time not knowing what would happen to him. It was him who the kidnapper freed eventually.
24	2	The kidnapper had held himself together and made his demands. It was the captive who he freed eventually.
24	3	The captive endured a long time not knowing what would happen to him. It was the kidnapper who freed him eventually.
24	4	The kidnapper had held himself together and made his demands. It was him who freed the captive eventually.

Conditions: 1, Patient given, Patient focussed; 2, Agent given, Patient focussed; 3, Patient given, Agent focussed; 4, Agent given, Agent focussed.

**Table A3:** **Experiment three**.

**Item**	**Condition**	**Story**	**Question**
1	1	The actor came to the end of a long day on the set. As he walked across to his trailer an officer arrested him.	What happened to the actor?
1	2	The officer began his shift late in the day. In the course of his long evening he arrested an actor.	What happened to the actor?
1	3	The actor came to the end of a long day on the set. As he walked across to his trailer an officer arrested him.	What did the actor do?
1	4	The officer began his shift late in the day. In the course of his long evening he arrested an actor.	What did the actor do?
2	1	The tramp searched the streets for a quiet place to weather the night. As he lay in an alley, a youth kicked him.	What happened to the tramp?
2	2	The youth wandered the streets looking to cause trouble. At the end of a quiet alley, he kicked a tramp.	What happened to the tramp?
2	3	The tramp searched the streets for a quiet place to weather the night. As he lay in an alley, a youth kicked him.	What did the tramp do?
2	4	The youth wandered the streets looking to cause trouble. At the end of a quiet alley, he kicked a tramp.	What did the tramp do?
3	1	The cowboy rode across the desert into the small dusty town. When he arrived a thief attacked him.	What happened to the cowboy?
3	2	The thief made her way through the sandstorm to a small town. When she arrived she attacked a cowboy.	What happened to the cowboy?
3	3	The cowboy rode across the desert into the small dusty town. When he arrived a thief attacked him.	What did the cowboy do?
3	4	The thief made her way through the sandstorm to a small town. When she arrived she attacked a cowboy.	What did the cowboy do?
4	1	The scientist spent the day categorizing all the new finds her team had made. When she thought she had finished, an assistant called her.	What happened to the scientist?
4	2	The assistant spend her day working on a the new finds from that week. She needed to check something so she called a scientist.	What happened to the scientist?
4	3	The scientist spent the day categorizing all the new finds her team had made. When she thought she had finished, an assistant called her.	What did the scientist do?
4	4	The assistant spend her day working on a the new finds from that week. She needed to check something so she called a scientist.	What did the scientist do?
5	1	The courier carried important packages across the wasteland, sometimes exposing him to danger. On his latest trip a hunter caught him.	What happened to the courier?
5	2	The hunter scoured the wastes for unsuspecting caravans and abandoned homes. During a recent trip he caught a courier.	What happened to the courier?
5	3	The courier carried important packages across the wasteland, sometimes exposing him to danger. On his latest trip a hunter caught him.	What did the courier?
5	4	The hunter scoured the wastes for unsuspecting caravans and abandoned homes. During a recent trip he caught a courier.	What did the courier?
6	1	The soldier served rather unwillingly in a multi-national corporation. As a result the manager fired him.	What happened to the soldier?
6	2	The manager ran his far-reaching company very strictly. Just the other day he fired an assistant.	What happened to the soldier?
6	3	The soldier served rather unwillingly in a multi-national corporation. As a result the manager fired him.	What did the soldier do?
6	4	The manager ran his far-reaching company very strictly. Just the other day he fired an assistant.	What did the soldier do?
7	1	The chemist enjoyed the scenery as he walked home from work. As he crossed a quiet road a motorist suddenly hit him.	What happened to the chemist?
7	2	The motorist drove along some of the quieter roads in the town. As he turned up the main street he suddenly hit a chemist.	What happened to the chemist?
7	3	The chemist enjoyed the scenery as he walked home from work. As he crossed a quiet road a motorist suddenly hit him.	What did the chemist do?
7	4	The motorist drove along some of the quieter roads in the town. As he turned up the main street he suddenly hit a chemist.	What did the chemist do?
8	1	The spy crawled through the vent into the heart of the dam complex. Just as he reached the control room a guard killed him.	What happened to the spy?
8	2	The guard prowled the metal hallways of the secret structure below the dam. As he entered the bottling room he killed a spy.	What happened to the spy?
8	3	The spy crawled through the vent into the heart of the dam complex. Just as he reached the control room a guard killed him.	What did the spy do?
8	4	The guard prowled the metal hallways of the secret structure below the dam. As he entered the bottling room he killed a spy.	What did the spy do?
9	1	The author had recently finalized a manuscript for submission. After some formal checks the published paid him.	What happened to the author?
9	2	The publisher had commissioned a new piece for his autumn releases. After receiving the manuscript he paid the author.	What happened to the author?
9	3	The author had recently finalized a manuscript for submission. After some formal checks the published paid him.	What did the author do?
9	4	The publisher had commissioned a new piece for his autumn releases. After receiving the manuscript he paid the author.	What did the author do?
10	1	The lawyer worked many difficult cases in the big commercial city. Just a few weeks ago, a killer hired him.	What happened to the lawyer?
10	2	The killer knew he was in trouble and needed to do something about it quickly. After some research, he hired a lawyer.	What happened to the lawyer?
10	3	The lawyer worked many difficult cases in the big commercial city. Just a few weeks ago, a killer hired him.	What did the lawyer do?
10	4	The killer knew he was in trouble and needed to do something about it quickly. After some research, he hired a lawyer.	What did the lawyer do?
11	1	The ninja could sneak through any room undetected, hiding in the shadows. Emerging through a doorway a sniper shot him.	What happened to the ninja?
11	2	The sniper lay silently on the rooftop, unmoved by the harsh weather. Peering then through his sights he shot a ninja.	What happened to the ninja?
11	3	The ninja could sneak through any room undetected, hiding in the shadows. Emerging through a doorway a sniper shot him.	What did the ninja do?
11	4	The sniper lay silently on the rooftop, unmoved by the harsh weather. Peering then through his sights he shot a ninja.	What did the ninja do?
12	1	The prisoner had remained in jail for many long years. At last his parole came through and the warden released him.	What happened to the prisoner?
12	2	The warden had run this jail for decades and had overseen many changes. Today a parole came through and he released a prisoner.	What happened to the prisoner?
12	3	The prisoner had remained in jail for many long years. At last his parole came through and the warden released him.	What did the prisoner do?
12	4	The warden had run this jail for decades and had overseen many changes. Today a parole came through and he released a prisoner.	What did the prisoner do?
13	1	The waiter had not worked at the restaurant for long, but suspicions had already arisen. Finally today the sheriff detained him.	What happened to the waiter?
13	2	The sheriff had almost finished a very long day, but had one last stop to make. At a new restaurant he detained a waiter.	What happened to the waiter?
13	3	The waiter had not worked at the restaurant for long, but suspicions had already arisen. Finally today the sheriff detained him.	What did the waiter do?
13	4	The sheriff had almost finished a very long day, but had one last stop to make. At a new restaurant he detained a waiter.	What did the waiter do?
14	1	The runner had many menial tasks and duties backstage at the concert. Today after several technical problems the singer pushed him.	What happened to the runner?
14	2	The singer became angry after technical problems had delayed their performance. As he stormed around backstage he pushed a runner.	What happened to the runner?
14	3	The runner had many menial tasks and duties backstage at the concert. Today after several technical problems the singer pushed him.	What did the runner do?
14	4	The singer became angry after technical problems had delayed their performance. As he stormed around backstage he pushed a runner.	What did the runner do?
15	1	The juror listened to a very difficult case which took weeks to conclude. In the closing days of the trial the defendant punched him.	What happened to the juror?
15	2	The defendant sat emotionless through most of his lengthy trial. As the conclusion drew near he punched a juror.	What happened to the juror?
15	3	The juror listened to a very difficult case which took weeks to conclude. In the closing days of the trial the defendant punched him.	What did the juror do?
15	4	The defendant sat emotionless through most of his lengthy trial. As the conclusion drew near he punched a juror.	What did the juror do?
16	1	The nurse spent her day off at home catching up on the TV shows she had missed. Right in the middle of a gripping episode the surgeon rang her.	What happened to the nurse?
16	2	The surgeon knew it would be a long shift as news of a terrible accident reached him. Extra staff were needed, so in between operations he rang a nurse.	What happened to the nurse?
16	3	The nurse spent her day off at home catching up on the TV shows she had missed. Right in the middle of a gripping episode the surgeon rang her.	What did the nurse do?
16	4	The surgeon knew it would be a long shift as news of a terrible accident reached him. Extra staff were needed, so in between operations he rang a nurse.	What did the nurse do?
17	1	The burglar hid the stolen items and tried to blend in with the other people in the street. After walking along a couple of streets a policeman trapped him.	What happened to the burglar?
17	2	The policeman heard that a robbery had taken place a few streets away. As he moved toward the area he trapped a robber.	What happened to the burglar?
17	3	The burglar hid the stolen items and tried to blend in with the other people in the street. After walking along a couple of streets a policeman trapped him.	What did the burglar do?
17	4	The policeman heard that a robbery had taken place a few streets away. As he moved toward the area he trapped a robber.	What did the burglar do?
18	1	The reporter tried desperately to hunt down a good story but without success. With such a poor performance the editor sacked him.	What happened to the reporter?
18	2	The editor enforced strict deadlines and demanded quality stories from all staff. After failing to meet the standard he sacked a reporter.	What happened to the reporter?
18	3	The reporter tried desperately to hunt down a good story but without success. With such a poor performance the editor sacked him.	What did the reporter do?
18	4	The editor enforced strict deadlines and demanded quality stories from all staff. After failing to meet the standard he sacked a reporter.	What did the reporter do?
19	1	The pupil disliked the lessons so she talked and laughed loudly. She caused disruption in the classroom and the teacher slapped her.	What happened to the pupil?
19	2	The teacher hoped to inform the unruly class about history and geography. Although she tried to stay calm she slapped a pupil.	What happened to the pupil?
19	3	The pupil disliked the lessons so she talked and laughed loudly. She caused disruption in the classroom and the teacher slapped her.	What did the pupil do?
19	4	The teacher hoped to inform the unruly class about history and geography. Although she tried to stay calm she slapped a pupil.	What did the pupil do?
20	1	The doctor had worked at the mental asylum for many years and was well known. During a routine checkup a patient murdered him.	What happened to the doctor?
20	2	The patient had resided at the mental hospital for many long years. During a normal monthly checkup he murdered a doctor.	What happened to the doctor?
20	3	The doctor had worked at the mental asylum for many years and was well known. During a routine checkup a patient murdered him.	What did the doctor do?
20	4	The patient had resided at the mental hospital for many long years. During a normal monthly checkup he murdered a doctor.	What did the doctor do?
21	1	The photographer had managed to capture some clear and compromising pictures. To avoid publication the celebrity compensated him.	What happened to the photographer?
21	2	The celebrity liked to control the pictures and information that magazines printed about her. To avoid a scandal, yesterday she compensated a photographer.	What happened to the photographer?
21	3	The photographer had managed to capture some clear and compromising pictures. To avoid publication the celebrity compensated him.	What did the photographer do?
21	4	The celebrity liked to control the pictures and information that magazines printed about her. To avoid a scandal, yesterday she compensated a photographer.	What did the photographer do?
22	1	The secretary had attended interviews at several businesses in the financial district. It was good news for her salary when a director appointed her.	What happened to the secretary?
22	2	The director found it very difficult to keep with his work when he had so much admin to attend to. Eventually he appointed a secretary.	What happened to the secretary?
22	3	The secretary had attended interviews at several businesses in the financial district. It was good news for her salary when a director appointed her.	What did the secretary do?
22	4	The director found it very difficult to keep with his work when he had so much admin to attend to. Eventually he appointed a secretary.	What did the secretary do?
23	1	The hiker spent his day moving up the mountain toward base camp. As he moved through an area of dense trees a hunter stabbed him.	What happened to the hiker?
23	2	The hunter had spent ages in the forest and began to get disorientated. In an automatic response to movement, he stabbed a hiker.	What happened to the hiker?
23	3	The hiker spent his day moving up the mountain toward base camp. As he moved through an area of dense trees a hunter stabbed him.	What did the hiker do?
23	4	The hunter had spent ages in the forest and began to get disorientated. In an automatic response to movement, he stabbed a hiker.	What did the hiker do?
24	1	The captive endured a long time not knowing what would happen to him. He guessed a ransom had arrived when the kidnapper freed him.	What happened to the captive?
24	2	The kidnapper had held himself together and made his demands. Though surprised that the random arrived, he freed the captive.	What happened to the captive?
24	3	The captive endured a long time not knowing what would happen to him. He guessed a ransom had arrived when the kidnapper freed him.	What did the captive do?
24	4	The kidnapper had held himself together and made his demands. Though surprised that the random arrived, he freed the captive.	What did the captive do?

Conditions: 1, Patient given, Patient-like; 2, Agent given, Patient-like; 3, Patient given, Agent-like; 4, Agent given, Agent-like.
